# Envafolimab plus lenvatinib and transcatheter arterial chemoembolization for unresectable hepatocellular carcinoma: a prospective, single-arm, phase II study

**DOI:** 10.1038/s41392-024-01991-1

**Published:** 2024-10-09

**Authors:** Yiwen Chen, Junlei Zhang, Wendi Hu, Xiang Li, Ke Sun, Yan Shen, Min Zhang, Jian Wu, Shunliang Gao, Jun Yu, Risheng Que, Yun Zhang, Fuchun Yang, Weiliang Xia, Aibin Zhang, Xiaofeng Tang, Xueli Bai, Tingbo Liang

**Affiliations:** 1 Department of Hepatobiliary and Pancreatic Surgery, The First Affiliated Hospital, Zhejiang University School of Medicine, Hangzhou, Zhejiang China; 2 Zhejiang Provincial Key Laboratory of Pancreatic Disease, Hangzhou, Zhejiang China; 3 Zhejiang Provincial Clinical Research Center for the Study of Hepatobiliary and Pancreatic Diseases, Hangzhou, Zhejiang China; 4 Cancer Center, Zhejiang University, Hangzhou, Zhejiang China; 5 Department of Pathology, The First Affiliated Hospital, Zhejiang University School of Medicine, Hangzhou, Zhejiang China

**Keywords:** Gastrointestinal cancer, Gastrointestinal cancer

## Abstract

Evidences regarding the feasibility of transcatheter arterial chemoembolization (TACE)-based therapy for unresectable hepatocellular carcinoma (uHCC) remains limited. This study aimed to investigate the efficacy and safety of TACE combined with envafolimab and lenvatinib for uHCC. Eligible patients with uHCC received envafolimab and lenvatinib after TACE until disease progression, conversion to surgery, intolerable toxicities, or death. The primary endpoint was the objective response rate (ORR) assessed according to Response Evaluation Criteria in Solid Tumors (RECIST) 1.1 criteria. Between March 2022 and July 2022, 38 patients were included for safety analysis, and 36 patients were included for efficacy analysis. As of the data cutoff (13 December 2023), the median follow-up was 16.9 months. The ORR was 50%, and disease control rate (DCR) was 83.3% per RECIST 1.1 (ORR and DCR of both 83.3% per modified RECIST (mRECIST)). The median progression-free survival (PFS) was 7.58 months. Of 36 patients, 17 patients were converted to resectable HCC with a surgical conversion rate of 47.2%, and 16 patients underwent surgery with R0 resection rate of 100%, pathologic complete response (pCR) rate of 31.3%. Overall incidences of treatment-related adverse events (TRAEs) of any grade was 97.4%. Grade ≥ 3 TRAEs were observed in 52.6% patients. No treatment-related deaths occurred. Image mass cytometry **(**IMC) analysis revealed that combined treatment improved the immune status of the tumor microenvironment, and resident macrophages had the potential to predict efficacy of this treatment. Envafolimab plus lenvatinib and TACE yielded promising survival outcomes and conversion efficiency with a tolerable safety profile. Trial registration Clinical trials: NCT05213221.

## Introduction

Hepatocellular carcinoma (HCC) is the third most common cause of cancer-related deaths worldwide, accounting for about 4.3% of all new cases and 7.8% of all cancer-related deaths.^1^ Hepatitis B virus (HBV) infection is a predominant risk factor for HCC, with a high incidence in China.^2^ Unfortunately, more than 50% of patients were diagnosed with intermediate or advanced disease, and had a poor prognosis with a 5-year overall survival (OS) of 3%.^3,4^ Considering that most patients with intermediate or advanced HCC are not suitable for radical resection, systemic therapy has been generally proposed to achieve tumor down-staging, conversion therapy, and long-term survival.^5,6^

Transcatheter arterial chemoembolization (TACE), recommended as the first-line treatment for intermediate/advanced HCC.^4,7^ However, repeated TACE may impair liver function and stimulate tumor angiogenesis by up-regulating the expression of vascular endothelial growth factor (VEGF), hence promoting tumor growth and metastasis.^8^ Besides, TACE may not always be curative, and most cases may eventually experience tumor recurrence.^9^ Thus, TACE plus anti-angiogenic therapy may harbor the potential to improve treatment effects compared with TACE alone. As evidenced by the TACTICS trial, TACE plus sorafenib significantly improved progression-free survival over TACE alone in patients with uHCC.^10^ Besides, improved clinical outcome was also observed from the combination of TACE and lenvatinib.^11^ However, the TACTICS trial failed to demonstrate long-term OS benefits (36.2 vs. 30.8 months).^10^ Overall, there is still an urgent need for better treatment strategies for uHCC.

Immune checkpoint inhibitors (ICIs) have revolutionized the treatment landscape of solid tumors and become a pivotal milestone in cancer treatment recently. Mechanically, the necrotic tumor tissues after TACE could significantly increase the abundance of CD4^+^ and CD8^+^ T cells and improve the immune microenvironment, triggering obvious tumor-specific immunological response and enhancing the clinical efficacy of TACE plus immunotherapies.^12^ Based on the synergistic mechanisms of anti-angiogenesis plus immunotherapies for uHCC,^13–16^ several studies demonstrated that the triple combination with TACE/anti-angiogenic therapies/immunotherapies could improve the long-term survival for uHCC patients.^17–21^ While most of the evidences were related to retrospective studies with relatively small sample size. More regrettably, there were not enough studies detailing the data of conversion surgery, which would be highly anticipated in future research.^22^ Thus, the ongoing investigation of a novel triple combination therapy to further improve the efficacy, safety, and resectability remained an essential unmet clinical need for uHCC.

Envafolimab was the world’s first subcutaneous single-domain anti-PD-L1 antibody and demonstrated promising efficacy in various advanced solid tumors,^23,24^ potentially serving as a more convenient and acceptable treatment regimen than those approved PD-1/PD-L1 inhibitors and revolutionizing the modes of immunotherapy.^25,26^ Taken together, we conducted a phase II study to evaluate the feasibility and safety of envafolimab plus lenvatinib and TACE in uHCC. In particular, we reported the outcomes of conversion surgery following our triple therapy, potentially expanding the active therapeutic strategy in intermediate to advanced uHCC.

Image mass cytometry (IMC) is an advanced technique that combines mass spectrometry and cytometry to visualize and measure the expression of multiple proteins at the single-cell level within tissue sections. This technology is particularly powerful in cancer research as it allows for the detailed analysis of the tumor microenvironment,^27^ which are crucial for understanding tumor progression, metastasis, and response to therapy. In this study, IMC was used to investigate why the combination therapy can improve patient prognosis and whether there are cell phenotypes that can predict treatment efficacy.

## Results

### Baseline characteristics

**Fig. 1:**
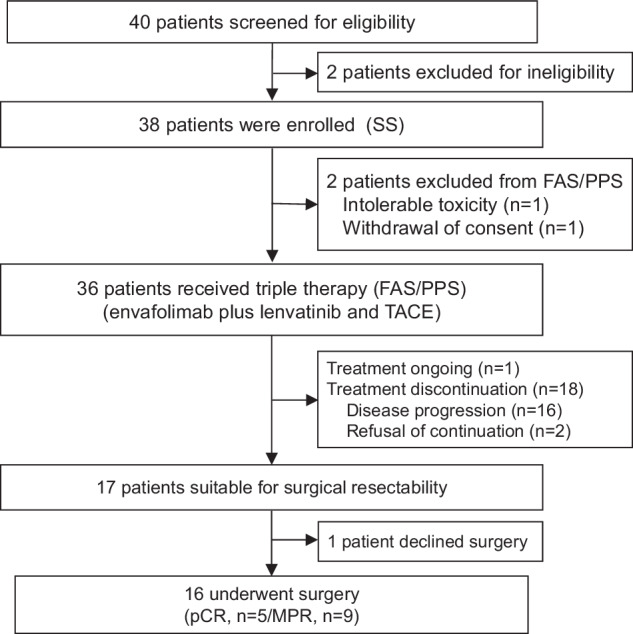
Flowchart of patient enrollment. Abbreviations: SS safety analysis set, FAS full analysis set, PPS per protocol set, TACE transhepatic arterial chemoembolization, pCR pathologic complete response, MPR major pathological response

Between March 2022 and July 2022, 40 patients with uHCC were screened for eligibility, and 38 patients were eligible for enrollment and received the protocol-specified therapy (Fig. 1). Therefore, 38 patients were included for safety analysis in the safety analysis set (SS), and 36 patients were included for efficacy analysis in the full analysis set (FAS). As of the cutoff date (13 December 2023), 1 patient continued therapy, and 20 patients (52.6%) discontinued treatment due to disease progression (*n* = 16), intolerable toxicity (*n* = 1), withdrawal of consent (*n* = 1), and refusal of continuation (*n* = 2). 17 patients were considered suitable for surgical resection, 16 patients of whom accepted the surgery.

The baseline demographics and disease characteristics were listed in Table 1. The median age of 38 patients was 55.5 years (range, 28–73 years), and most of them were male (81.6%) with BCLC stage C (55.3%). In 21 patients with BCLC C stage, 13 of them were with only PVTT, 1 patient was with both PVTT and HVTT, 4 patients were with only HVTT, and 3 patients were with extrahepatic metastasis. In addition, there were 18 patients with IIIa, 3 patients with IIIb in 21 patients with BCLC C stage. At baseline, all patients were reported as Child-Pugh class A with Child-Pugh scores of 5 (94.7%) or 6 (5.3%), and with a median ALBI score of −2.78 (range, −3.44 to −2.03). At study enrollment, 3 (7.9%) patients had extrahepatic metastases, and 7 (18.4%) patients had received prior treatments (3 (7.9%) surgery and 4 (10.5%) TACE).

**Table 1:** Patient baseline characteristics

Characteristics	All patients (*n* = 38)
Age (years), median (range)	55.5 (28–73)
Sex, *n* (%)	
Male	31 (81.6%)
Female	7 (18.4%)
BMI, median (range)	23.9 (17.7-29)
Etiology, *n* (%)	
Hepatitis B	29 (76.3%)
Alcohol	3 (7.9%)
Schistosome	2 (5.3%)
Unknown	4 (10.5%)
ECOG-PS, *n* (%)	
0	38 (100.0%)
Child-Pugh score, *n* (%)	
5	36 (94.7%)
6	2 (5.3%)
ALBI Score, median (range)	−2.78 (−3.44, −2.03)
ALBI grade	
1	24 (63.2%)
2	14 (36.8%)
CNLC staging, *n* (%)	
IIa	4 (10.5%)
IIb	13 (34.2%)
IIIa	18 (47.4%)
IIIb	3 (7.9%)
BCLC staging, *n* (%)	
B	17 (44.7%)
C	21 (55.3%)
PVTT, *n* (%)	
Vp1	1 (2.6%)
Vp2	4 (10.5%)
Vp3	9 (23.7%)
Absent	24 (63.2%)
HVTT, *n* (%)	
Vv1	2 (5.3%)
Vv2	3 (7.9%)
Absent	33 (86.8%)
Tumor burden score (TBS), *n* (%)	
<8	16 (42.1%)
≥8	22 (57.9%)
Extrahepatic metastasis, *n* (%)	3 (7.9%)
Prior treatment, *n* (%)	7 (18.4%)
Surgery	3 (7.9%)
TACE	4 (10.5%)

*ECOG-PS* Eastern Cooperative Oncology Group performance status, *ALBI* albumin-bilirubin, *CNLC* China Liver Cancer, *BCLC* Barcelona Clinic Liver Cancer, *TACE* transhepatic arterial chemoembolization, *BMI* body mass index, *PVTT* portal vein tumor thrombosis, *HVTT* hepatic vein tumor thrombosis

### Efficacy

**Fig. 2:**
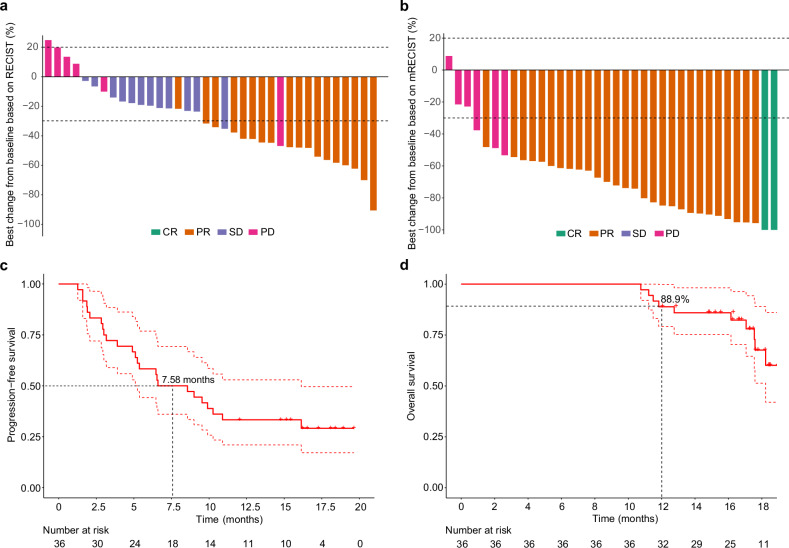
Tumor response and survival outcomes. **a** Waterfall plot of the best percentage change in target lesion size from baseline according to RECIST 1.1; (**b**) Waterfall plot of the best percentage change in target lesion size from baseline according to mRECIST; (**c**) Kaplan–Meier curves of progression-free survival; (**d**) Kaplan–Meier curves of overall survival. RECIST Response Evaluation Criteria in Solid Tumors, mRECIST Modified Response Evaluation Criteria in Solid Tumors, CR complete response, PR partial response, SD stable disease, PD progressive disease, Mo months

As of the data cutoff (13 December 2023), the median follow-up was 16.9 months (IQR, 14.8–18.1 months). The median duration of lenvatinib was 3.17 months (IQR, 2.07-4.47 months). The median cycles of TACE were 2 (IQR, 1–2), and the median cycles of envafolimab were 4 (IQR, 2.75–6). The objective response rate (ORR) per Response Evaluation Criteria in Solid Tumors (RECIST) 1.1 was observed in 18 patients (50%; 95% confidence interval (CI): 32.9–67.1%) in the FAS population, meeting the primary endpoint (Fig. 2a and supplementary Table 1). A total of 12 (12/36, 33.3%) patients achieved stable disease (SD) as their best response. Thus, the disease control rate (DCR) was 83.3% (95% CI: 67.2–93.6%) in this population. The median duration of response (DoR) per RECIST 1.1 was 6.8 months (95% CI: 2.77 to not available (NA)). In the supportive analysis from the per protocol set (PPS) population, the ORR was 50.0% (95% CI: 32.9–67.1%), and DCR was 83.3% (95% CI: 67.2–93.6%) as per RECIST 1.1, respectively, which were consistent with results from FAS analysis.

As per modified RECIST (mRECIST), 2 patients (5.6%) achieved complete response (CR), and 28 (77.8%) patients attained partial response (PR) with no SD and 6 progressive disease (PD), leading to both ORR and DCR of 83.3% (95% CI: 67.2–93.6%) (Fig. 2b and supplementary Table 1). The mDoR per mRECIST was 8.12 months (95% CI: 4.9 to NA). The response rate in the PPS population per mRECIST was supportive of the primary analysis by RECIST 1.1 with ORR and DCR of both 83.3% (95% CI: 67.2–93.6%). The median PFS (mPFS) was 7.58 months (95% CI: 5.1 to 16.1) per RECIST 1.1 or mRECIST (Fig. 2c). The median OS (mOS) was 19.9 months (95% CI: 18.2 to NA) with 1-year OS of 88.9% (95% CI: 79.2–99.8%) (Fig. 2d).

### Surgery outcomes

Details of surgery outcomes were as follows. A total of 17 patients were assessed as being suitable for surgery after conversion therapy, resulting in a surgical conversion rate of 47.2% (17/36). 16 patients of whom accepted the surgery and 1 patient declined surgery. In the surgical population, all of the patients (16/16, 100%) achieved an R0 resection, and nine (9/16, 56.3%) patients had a major pathological response (MPR), including five (5/16, 31.3%) with pathologic complete response (pCR). The 1-year disease-free survival (DFS) rate was 56.2% (95% CI: 36.5–86.7%). With regard to postoperative prognosis, the 1-year disease-free survival (DFS) rate of patients with pCR was significantly better than those without pCR (100% vs 36.4%; *p* < 0.05) in this trial. In addition, postoperative follow-up is still ongoing, and postoperative prognosis results will be further upgraded in the future.

### Safety

Table 2 summarized treatment-related adverse events (TRAEs) reported by 10% or more of patients and all reported grade 3 or worse TRAEs. Of 38 patients, 37 (97.4%) experienced at least one TRAE (all causality) of any grade, with TRAEs of grade ≥3 being reported in 20 (52.6%) patients. The most common grade 3 or worse TRAEs were increased aspartate aminotransferase (AST) (34.2%), and increased alanine aminotransferase (ALT) (18.4%), and decreased platelet count (15.8%). Increased AST and ALT were thought mainly caused by the TACE. During the entire study process, only one (2.6%) patient experienced serious AE (SAE; gastrointestinal bleeding), and required treatment discontinuation. No treatment-related deaths occurred.

**Table 2:** Treatment-related adverse events

	All grades	Grade 3 or worse
Any events, *n* (%)	37 (97.4)	20 (52.6)
Frequent events, *n* (%)		
Aspartate aminotransferase increased	28 (73.7)	13 (34.2)
Alanine aminotransferase increased	25 (65.8)	7 (18.4)
White blood cell decreased	16 (42.1)	0
Platelet count decreased	15 (39.5)	6 (15.8)
Neutrophil count decreased	15 (39.5)	1 (2.6)
Gastrointestinal bleeding	16 (42.1)	1 (2.6)
Thyroid stimulating hormone increased	14 (36.8)	0
Hypoalbuminemia	11 (28.9)	0
Endocrine disorders	8 (21.1)	0
Weight loss	7 (18.4)	0
Blood bilirubin increased	5 (13.2)	0
Rash	5 (13.2)	0
Hypothyroidism	6 (15.8)	0
Hyperthyroidism	4 (10.5)	0
Palmar-plantar erythrodysesthesia syndrome	3 (7.9)	2 (5.3)
Hypertension	2 (5.3)	1 (2.6)
Hypertriglyceridemia	1 (2.6)	1 (2.6)

### Combination therapy may enhance immunity

**Fig. 3:**
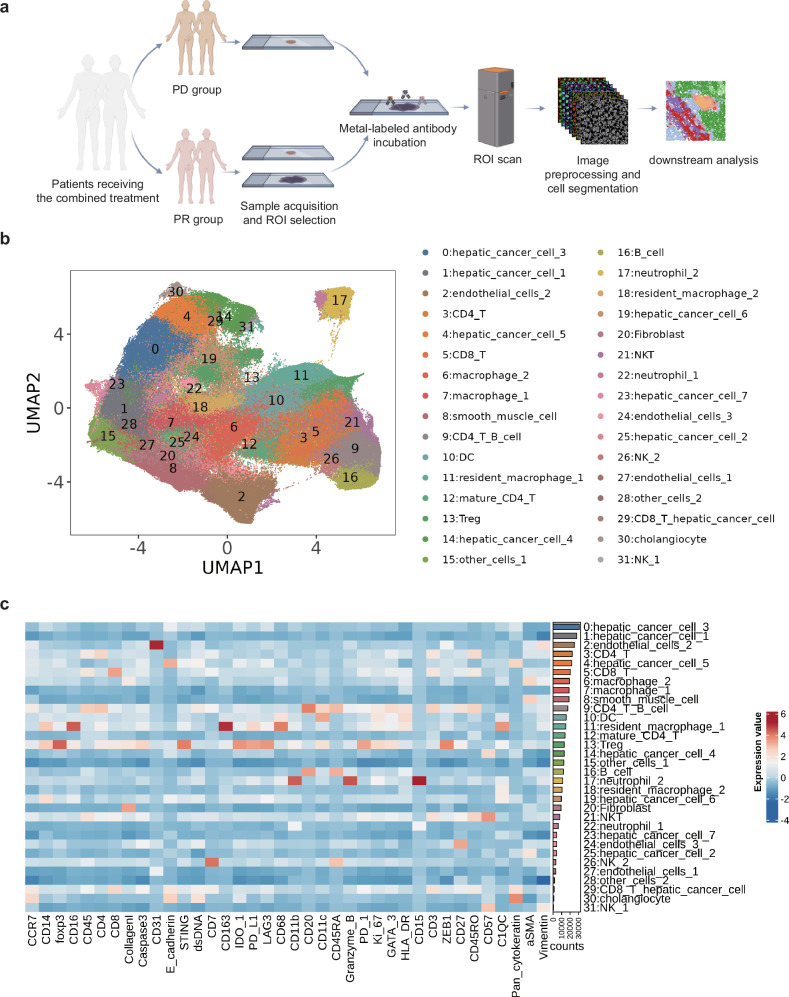
Analysis workflow and clustering dimensionality reduction results. **a** Flowchart of sample analysis. **b** Uniform manifold approximation and projection (UMAP) plot of single-cell clustering after segmentation of image mass cytometry (IMC) scanned images. **c** Heatmap showing the expression levels of various markers for each cell cluster

The details and flowchart of IMC analysis were shown in Fig. 3a. As shown in Fig. 3b, a total of 31 cell populations were identified, including those constituting the main tissue structures: hepatic cancer cells (group 0, 1, 4, 14, 19, 23, 25), endothelial cells (group 2, 24, 27), smooth muscle cells (group 8), fibroblasts (group 20), and cholangiocyte (group 30). We also identified several kinds of major lymphoid immune cells: CD4^+^ T cells (group 3, 12), CD8^+^ T cells (group 5), B cells (group 16), regulatory T cells (Treg, group 13), NK cells (group 26, 31), and NKT cells (NKT, group 21). Additionally, we identified major myeloid immune cells: neutrophils (group 17, 22), dendritic cells (DC, group 10), resident macrophages (group 11, 18), and other macrophage groups (group 6, 7). Furthermore, we observed that group 9 exhibited simultaneous expression of CD20^+^ (a B-cell marker) and CD3^+^/CD4^+^ (CD4^+^ T-cell markers). This could be attributed to the dense distribution of B cells and CD4^+^ T cells within the tertiary lymphoid structures (TLS). The protein expression of markers for each cell population was displayed in the heatmap in Fig. 3c. However, due to the limited resolution of IMC scanning, some cell membrane markers were not completely separated into two distinct cells during cell segmentation (Supplementary Fig. 1).^27^ And some representative IMC images and corresponding H&E images are displayed in supplementary Fig. 1b.

**Fig. 4:**
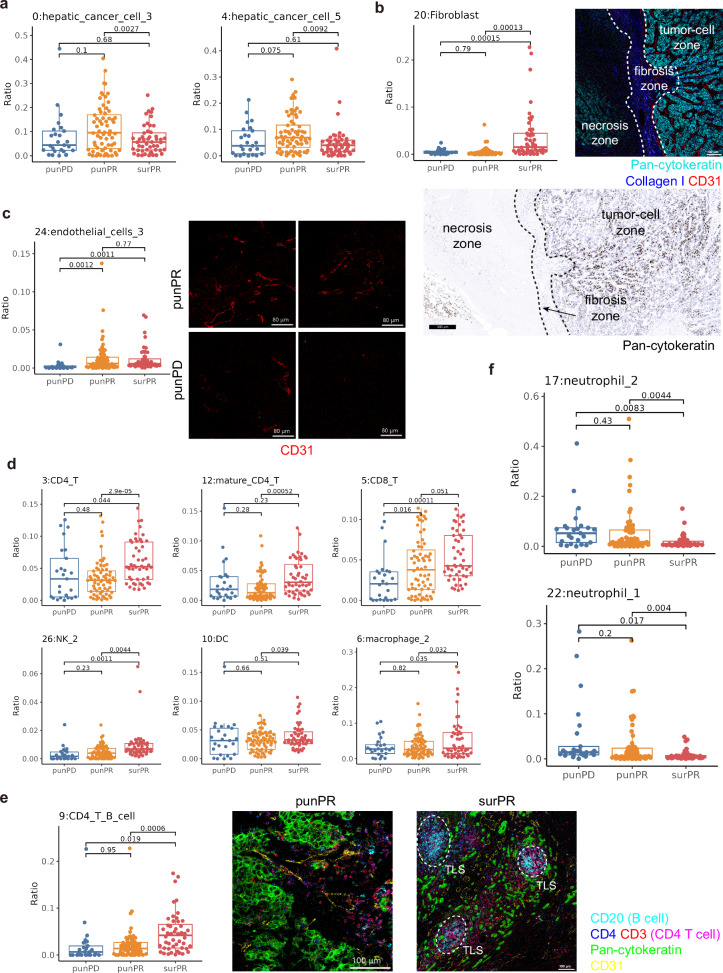
Statistical differences in immune cells among three groups. **a** Statistical graph of immune cells in liver cancer cells. **b** Left: Statistics of fibroblasts; Right: fibrotic barrier formed between tumor tissue and necrotic tissue, with the image mass cytometry (IMC) image on the top showing Pan-cytokeratin (cyan), Collagen I (blue), CD31 (red), scale bar, 100 μm; and the immunohistochemistry image of Pan-cytokeratin at the bottom, scale bar, 300 μm. **c** Statistics of endothelial cells on the left, with the representative IMC image of endothelial cells on the right, scale bars, 80 μm. **d** Statistical graph of various immune cell clusters. **e** Left: Statistics of CD4 + T cells with B cells in TLS; Right: Representative IMC image of TLS, with CD20 + B cells (cyan), CD4 (blue), CD3 (red), CD4 + T cells represented in pink after overlap of CD4 and CD3, Pan-cytokeratin (green), CD31 (yellow), TLS outlined by white dashed lines. Scale bars, 100 μm. **f** Statistical graph of neutrophils

Subsequently, we performed statistical analysis on these 31 cell populations in the three groups and observed that two groups of hepatic cancer cells (group 0, 4) significantly decreased in PR patients after treatment (Fig. 4a). Additionally, we observed a significant increase in fibroblasts after treatment, which may be associated with the formation of fibrous barriers between tumor cells that have undergone TACE-induced cell death and normal tumor cells or hepatocytes (Fig. 4b). Based on the analysis of the cellular composition of the tissues, we found that the pre-treatment samples of endothelial cells in PD patients showed significantly lower numbers compared to the pre-treatment and post-treatment samples of PR patients (Fig. 4c). This observation suggested that the limited presence of endothelial cells may hinder the delivery of drugs to the tumor site, thereby leading to a poorer prognosis. Importantly, this finding has the potential to serve as a significant prognostic factor for predicting patient outcomes.

Interestingly, significant improvements in the immune environment of PR patients’ tissues after treatment were also observed. Various immune cell populations showed a notable increase, including CD4^+^ T cells (group 3, 12), CD8^+^ T cells (group 5), NK cells (group 26), DC (group 10), and macrophage groups (group 6) (Fig. 4d). Additionally, there was a significant increase in cells from group 9 that co-expressed B cells and CD4^+^ T cells in surPR patients, indicating a potential increase in TLS in the tissues after treatment (Fig. 4e). Conversely, immune suppressive cells such as tumor-associated neutrophils exhibited a significant decrease (group 17, 22) (Fig. 4f). In summary, the analysis of the immune microenvironment effectively demonstrated the profound impact of our combination therapy on immune modulation. While effectively eliminating a large number of tumor cells, the treatment with immune checkpoint blockade (ICB) also greatly activated the tumor’s immune state, providing a secondary hit to enhance tumor cell killing.

### Resident-macrophage enriched cellular neighborhood was predictive of treatment efficacy

According to our previously established method,^28^ we captured the cellular neighborhood (CN) context by constructing windows around each cell, including the 20 closest cells (Supplementary Fig. 2a). Subsequently, based on the predominant cell populations enriched within each CN, we defined the following CN categories: neutrophil enriched (CN1), hepatic cancer cell-enriched (CN2, CN3, CN5, CN10), TLS (enriched in CD4^+^ T cells and B cells, CN4), resident macrophage enriched (CN6), vascular zone (enriched in endothelial cells and smooth muscle cells, CN7), T cell enriched (CN8), and macrophage and fibroblast enriched (CN9) (Supplementary Fig. 2b). Then, we assigned colors to the defined CNs and projected them back onto the spatial context, resulting in a CN map that corresponded well with the original IMC images, such as the vascular zone and TLS region (Supplementary Fig. 2c).

Subsequently, we performed statistical analysis on the CNs, which aligned with the previous single-cell analysis (the analysis of cells after single-cell segmentation and does not include their spatial information). The hepatic cancer cell enriched CNs (CN2, CN10) were significantly decreased in the post-treatment samples of PR patients (Supplementary Fig. 2d). The macrophage and fibroblast enriched CN9 showed a significant increase. Based on the image correspondence, we identified this region as the fibrous tissue at the interface of tumor cells and immune cells, which frequently appeared between necrotic and normal tumor tissues. The macrophages within this CN were found to be interspersed among fibroblasts, and their roles in the tumor remained to be investigated (Fig. 4b and supplementary Figs. 2e and 3).

The most notable changes were observed in the TLS-related CNs (CN4), consistent with the single-cell analysis. This region significantly increased in PR patients after treatment, indicating a substantial improvement in the immune environment. This finding was highly relevant because TLS had a strong association with patient prognosis (Supplementary Fig. 2f). In the analysis of immune suppressive regions, we observed a significant decrease in the neutrophil-enriched CN in PR patients after treatment (Supplementary Fig. 2g). Furthermore, a significant difference was observed in the resident macrophage enriched CNs between pre-treatment PR and PD patients. This cell population is known to promote liver cancer growth,^28^ highlighting the strong predictive value of this CN in the pre-treatment setting (Supplementary Fig. 2h). In conclusion, the CN analysis confirmed that the combination therapy improved the immune microenvironment in PR patients from a spatial perspective. Additionally, it revealed the significant predictive potential of the resident macrophage-enriched CN for patient response to this treatment.

## Discussion

To our best knowledge, this was the first prospective phase II study to evaluate the feasibility of triple therapy with envafolimab plus targeted therapy and TACE in uHCC. Our regimen demonstrated promising response with a manageable safety profile. Besides, this study also showed an encouraging surgical conversion rate of 47.2% and pathological outcomes with pCR of 31.3%. In brief, this triple regimen followed by surgery might represent a novel beneficial treatment option for intermediate-advanced uHCC.

Generally, curative conversion could be feasible with the optimal timing in uHCC patients achieving a deep tumor response after systemic therapy,^29^ potentially prolonging the survival.^22^ In contrast, our regimen achieved a numerically higher response rate per either RECIST 1.1 (50%) or mRECIST (83.3%) compared with several studies.^11,30^ Notably, tumor response here outperformed the result of the IMbrave150 trial, either in the total population (47.4% vs. 30%) or in patients with BCLC stage B (94.12% vs.44%) or C disease (66.67% vs.27%).^31^ These suggested that our triple combination may harbor the therapeutic potential to be formulated into conversion therapy for advanced uHCC beyond intermediate-stage patients. When compared with the similar trials (Supplementary Table 4),^19,20,31,32^ the mPFS observed in this study was comparatively shorter. We noticed that the exclusion criteria on cutoff value of tumor burden in this study was relatively higher (70% vs. 50%), as a result, patients may have higher tumor burden in baseline condition. Besides, the treatment methods, such as the sorts of TKI and PD-1/PD-L1 antibody, or the type of TACE approaches (with lipiodol or drug-eluting beads) may also affect the survival, which needs further exploration in the future. Importantly, we reasoned that the OS improvement would be compared with other studies as the follow-up continued, given the evidence of OS (22-23.6 months) in several studies of immunotherapy plus lenvatinib and TACE.^17,33^ The true effects of this triple therapy on long-term survival remained to be further scrutinized in the final analysis.

Accumulating studies have evaluated the feasibility of different combination therapies as conversion therapy in uHCC and demonstrated surgical conversion rates of ~15–37.5%.^34–36^ By contrast, we reported a higher conversion resection rate of 47.2%. In parallel, patients with surgery achieved promising pathological outcomes with a pCR of 31.3%, MPR of 56.3%, and R0 resection rate of 100%, which was also comparable with or outperformed results from other triple combinations.^35^ However, cross-trial comparisons should be interpreted with caution owing to the potential difference in the study designs and the criteria for surgical resection. Despite the potential of this triple regimen as conversion therapy, accurate results concerning OS and postoperative DFS should be scrutinized in the future.

Combining envafolimab with lenvatinib on top of TACE was clinically safe; the AE spectrum of this triple combination was consistent with the known safety profile of each agent,^37,38^ and no new or unexpected safety signals were identified in this study. The rate of any grade (97.5% vs. 98.2–99%) and grade 3 or worse (55.0% vs. 61.1–68%) TRAEs observed here was numerically lower than that in the IMbrave 150 trial and envafolimab study,^37,39^ indicating that our regimen did not increase the risk of toxicity events. Only one (2.5%) patient discontinued therapy due to serious gastrointestinal bleeding, and no treatment-related deaths occurred. Moreover, no infusion reactions were reported, which may be ascribed to the subcutaneous administration mode of envafolimab differing from other PD-1/PD-L1 inhibitors.^26^

IMC analysis provided insights into the immune microenvironment changes in patients with HCC undergoing combination therapy. One of the key findings in this study was the significant decrease of certain populations of hepatic cancer cells after treatment, which may be attributed to the cytotoxic effects of the combined treatment on tumor cells.^40,41^ Additionally, we observed the increase of fibroblasts, which may be associated with the formation of fibrous barriers between tumor cells that underwent TACE-induced cell death and normal tumor cells or hepatocytes.^42,43^ In brief, these findings highlighted the efficacy of the combination therapy in targeting and reducing tumor burden. Furthermore, a significant increase of various types of immune cell populations was observed after treatment, suggesting that the combination therapy not only eliminated tumor cells, but also activated the immune system, leading to enhanced tumor cell killing. TLS are known to play a crucial role in anti-tumor immune responses and are associated with better patient outcomes.^44–46^ We also observed an increase of cells co-expressing B-cell and CD4^+^ T-cell markers, indicating the potential formation of TLS in the tissues after treatment. Another significant finding was the limited presence of endothelial cells in pre-treatment samples of non-responding patients (PD group) compared to pre-treatment and post-treatment samples of responding patients (PR group), which suggested that the presence of endothelial cells may play roles in drug delivery to the tumor site and could serve as a prognostic factor for patient outcomes. The spatial analysis of the immune microenvironment further supported our findings from the single-cell analysis. We found that the changes observed in these CNs after treatment were aligned with the improvements in the single-cell analysis. Notably, the increase of TLS-related CNs in PR patients after treatment reinforced the significance of TLS in the immune response against tumors.

This study had several limitations. First, this was a single-arm study with a relatively small sample size. In the future, more randomized controlled trials (RCTs) with larger sample size should be further performed. Second, the follow-up period of this study was relatively short. Third, this study was limited by the absence of quality-of-life (QoL) data, which could not examine this combination strategy on symptoms of intermediate-advanced uHCC. Fourth, further evaluation of biomarkers for predicting efficacy is warranted in a larger cohort.

In conclusion, envafolimab plus lenvatinib and TACE exhibited favorable efficacy and a manageable safety profile. More importantly, this combination therapy resulted in tumor downsizing, and allowed uHCC patients to have access to conversion surgery with favorable R0 resection rate and pCR rate, potentially representing a novel valuable treatment option for this population in the clinical setting.

## Materials and methods

### Patients

This prospective, open-label, single-arm phase II study was conducted at the First Affiliated Hospital of Zhejiang University School of Medicine in February 2022 to investigate the efficacy and safety of envafolimab plus lenvatinib and TACE in intermediate to advanced uHCC. Eligible patients with uHCC were consecutively enrolled in this study. The inclusion criteria of this study were as follows: (1) Age between 18 and 75 years old; (2) Imaging, histologically, or cytologically confirmed HCC (based on the American Association for the Study of Liver Diseases (AASLD) criteria);^47^ (3) Barcelona Clinic Liver Cancer stage B or C;^4^ (4) Confirmed uHCC by the experienced hepatobiliary pancreatic (HBP) multidisciplinary team (MDT), which has been established by our center since 2007;^48^ (5) Had at least one measurable lesion defined by the Response Evaluation Criteria in Solid Tumors (RECIST) 1.1;^49^ (6) No prior systemic anti-tumor therapies; (7) Eastern Cooperative Oncology Group performance score (ECOG-PS) of 0-1;^50^ (8) Child-Pugh score of ≤ 7; (9) Adequate organ functions and predicted life expectancy of at least 3 months. Key exclusion criteria included: tumor burden exceeding 70% of the whole liver, main trunk portal vein tumor thrombus (Vp4), immune deficiency, and central nervous system metastasis. Besides, full eligibility criteria were listed in supplementary Table 2.

### Ethics statements

All patients provided written informed consent before enrollment. This study was approved by the Clinical Research committee of the First Affiliated Hospital of Zhejiang University School of Medicine (Ethical approval number, LunShen [2021] No. 93), and conducted per the Declaration of Helsinki, the International Conference on Harmonization (ICH), Good Clinical Practice guidelines (GCP) guidelines, and applicable laws and regulations. This research has been registered on ClinicalTrials.gov with the identifier of NCT05213221.

### Procedures

#### TACE procedure

TACE was conducted as described.^10^ TACE was repeated every 6 weeks based on the “on-demand” principle according to the follow-up results and investigators’ evaluation (mainly based on the proportion of viable tumors or lesion reduction of less than 50% from the baseline).

#### Systemic treatment of envafolimab/lenvatinib

Patients received combination therapy of envafolimab (300 mg, subcutaneous injection (sc), Q3W) and lenvatinib (12 mg (≥60 kg) or 8 mg (<60 kg) orally, once daily) from the day of the first TACE until disease progression, conversion to surgery, intolerable toxicities, withdrawal of consent, or death occurred. Dose reduction of envafolimab was not allowed during this study, while dose interruptions or discontinuation was permitted based on the severity of adverse events (AEs). Dose modification, interruption, or discontinuation for lenvatinib was applicable based on the severity of AEs. Detailed dose modification criteria for these two agents were listed in supplementary Table 3.

#### Surgery

Criteria for resectability were determined by the MDT during the radiological evaluation of tumor response. Criteria for resectability were as follows: (1) A Child-Pugh score ≤ 7, an indocyanine green retention rate at 15 min (ICG R15) ≤ 10%, and an ECOG-PS of 0-1; (2) Tumor lesions experiencing CR or PR based on the mRECIST; (3) Patients without liver cirrhosis having a remnant liver volume ≥ 30% of standard liver volume, or patients with liver cirrhosis having a remnant liver volume ≥45% of standard liver volume; (4) Inactivation and regression of vascular tumor thrombi, and technically resectable with an aim to achieve R0 resection; (5) Absence of other contraindications for surgery. The surgical approach was performed on medically fit patients as previously reported.^51^ In addition, patients who did not meet the criteria for surgery continued to receive the combination therapy until disease progression, intolerable toxicity, etc.

### Outcomes and assessment

The primary endpoint was the ORR per RECIST 1.1, which was defined as the percentage of patients achieving CR and PR. The secondary endpoints included DCR (defined as the proportion of patients with CR, PR, and SD), DoR (defined as the time from first documented evidence of CR or PR to PD or death from any cause, whichever occurred first), PFS (defined as the time from first treatment to the PD or death or recurrence from any reason), and OS (defined as the time from date of the first treatment to the date of death of any reason or the date of the last follow-up). DCR, DoR, and PFS were evaluated using both RECIST 1.1 and mRECIST. Other endpoints included surgical conversion rate, pCR (with definition of the complete absence of residual viable tumor cells after HE staining from the completely resected specimen) rate, R0 resection rate, MPR (defined as ≤10% of viable tumor cells from resected specimen) and DFS (defined as the duration from time of surgery to the first occurrence of local or distant recurrence or death).

Tumor assessments were performed via enhanced computed tomography (CT) or magnetic resonance imaging (MRI) at baseline and every 6 weeks (±7 days) during the study until imaging-based progression, the start of new anti-tumor treatments, withdrawal of consent, loss to follow-up, or deaths, whichever occurred first. Safety was assessed by the physical examination, vital signs, hematological and biochemical laboratory tests, and the incidences of AEs from the start of the study treatment until 60 days after the last administration or until the end of the study. TRAEs were graded according to the National Cancer Institute Common Terminology Criteria for Adverse Events (NCI-CTCAE) version 5.0.

### Image mass cytometry analysis

#### IMC image preprocessing and cell segmentation

In order to elucidate why the combination therapy of TACE, envafolimab, and lenvatinib can improve patient prognosis, the changes in the tumor immune microenvironment of patients with HCC were reviewed and analyzed by IMC analysis. Patients who achieved PR per RECIST 1.1 as the best response were included in the PR group, and who achieved PD per RECIST 1.1 as the best response were included in the PD group. In addition, some patients received biopsy of the tumor before the treatment. The biopsy samples obtained from the patients of PR group were labeled as punPR, and punPD for that of the patients of PD group. In PR group, some patients received surgical resection. And the paraffin samples from these patients were labeled as surPR. Subsequently, we performed hematoxylin and eosin (HE) staining on the sections and identified regions of interest (ROI) with portal areas or a higher density of immune cell infiltration. We stained adjacent sections with 38 metal-conjugated antibodies and performed IMC scanning on the corresponding ROI. Subsequently, we segmented the scanned images into single cells and conducted a series of analyses, including clustering and dimension reduction using our previously established methods.^28^

The procedures for analyzing data from IMC encompassed a sequence of four distinct phases: correction of overlapping signals, refinement of image quality, enhancement of image clarity, and delineation of individual cells. For the overlapping signals within each channel, we employed a previously established spillover matrix.^52^ During the refinement stage, we utilized a median filter technique to reduce noise, selecting a 3 × 3 window size so that the median value within the 3×3 area around each pixel was used as the output.^53^ To improve the visual sharpness of the images, we adjusted the intensity levels using a function that optimized the spread of intensity values across the full potential range, specifically from 0 to 255. For the purpose of distinguishing cells or elements across the various IMC channels, a segmentation method that could take connectivity into account was adopted.^54^ This involved using a function to identify connected components for cell delineation. Regarding the channels that detected membranes, we discarded any artifacts that were positioned more than 15 pixels away from the nearest nucleus center. All of these processes were conducted using MATLAB version R2017a, with the code available publicly at the provided GitHub repository.

#### IMC downstream analysis

In the subsequent stages of IMC data analysis, we normalized marker expression to the 99th percentile for each channel on a cell-by-cell basis. To rectify batch variations, we implemented the Harmony package (version 0.1.0).^55^ For cellular clustering, we used an R version of the algorithm PhenoGraph, setting the parameter to 100 nearest neighbors, and depicting the cluster averages in a heatmap for further interpretation. We captured the CN context by constructing windows around each cell, including the 20 closest cells based on Euclidean distance, and then applied K-means clustering to categorize the windows based on the composition of 31 identified cell types. With a chosen value of *k* = 15, each cell was associated with a neighborhood cluster based on its surrounding window. The accuracy of neighborhood assignments was confirmed by superimposing Voronoi diagrams onto the original tissue IMC images.

### Spatial analysis

In order to explore the dynamics between cells, we utilized a permutation-based testing approach, specifically the interactions function from the imcRtools package, version 1.0.2.^56^ This method assessed whether certain cell types within a given CN engaged or avoided each other would be expected by chance. We compiled a multidimensional array for each individual by tabulating the occurrence of each cell type within every CN, thereby generating a patient-specific distribution tensor. Subsequently, this array was categorized according to patient groups, namely punPD, punPR, and surPR.

### Statistical analysis

Sample size calculation was performed using PASS 15.0. We hypothesized an increase of ORR (per RECIST 1.1) from 27.8% (historical data of lenvatinib plus TACE^57^) to 50% of envafolimab plus lenvatinib and TACE. A sample size of 35 patients would provide at least 80% power and a two-sided α of 0.05. Considering an approximate dropout rate of 10%, 39 patients would be enrolled in this study.

The efficacy analysis was conducted primarily in the FAS, which included all enrolled patients who received at least one dose of study treatment and met the eligibility criteria (those patients who canceled consent or were lost to follow-up before the first imaging evaluation were excluded from FAS population). Efficacy data were also analyzed in the PPS population as supportive results. The PPS should have measurable lesions at baseline, at least one post-baseline radiological evaluation, no major protocol violations, and good compliance. Safety was assessed in the SS, which included patients who met the eligibility criteria, received at least one dose of study treatment, and had safety records.

Patient characteristics, safety outcomes, and tumor responses were summarized descriptively. Descriptive measures for continuous variables were median (interquartile range (IQR)) or range, and for categorical variables were frequencies (percentage (%)). 95% CI of the ORR and DCR were calculated using the Clopper-Pearson method based on the binomial distribution. PFS and OS were estimated using the Kaplan-Meier method in this study. Survival data from patients without disease progression or death were censored at the date of their last tumor assessment.

All of the statistical analyses were performed using SAS software (version 9.4; SAS Institute, Cary, NC, USA), and a two-sided *p*-value of <0.05 was considered statistically significant.

## Supplementary information

Supplementary Materials

Figure S1

Figure S2

Figure S3

## Data Availability

The datasets in this study are available upon request from the corresponding author. The IMC data reported in this paper have been deposited in the OMIX, China National Center for Bioinformation/Beijing Institute of Genomics, Chinese Academy of Sciences (https://ngdc.cncb.ac.cn/omix: accession no.OMIX007474).
