# Supplementary material for: Envafolimab plus lenvatinib and transcatheter arterial chemoembolization for unresectable hepatocellular carcinoma: a prospective, single-arm, phase II study
Source: Signal Transduct Target Ther. 2024 Oct 9;9:280. doi: 10.1038/s41392-024-01991-1 (PMC11464841; doi:10.1038/s41392-024-01991-1)
Supplement: Supplementary file 1 — Supplementary Materials [file 41392_2024_1991_MOESM1_ESM.docx]

Supplementary Materials for

Envafolimab plus lenvatinib and transcatheter arterial chemoembolization for unresectable hepatocellular carcinoma: a prospective, single-arm, phase II study

Yiwen Chen^1, 2, 3, 4^, Junlei Zhang^1, 2, 3, 4^, Wendi Hu^1^, Xiang Li^1, 2, 3, 4^, Ke Sun^5^, Yan Shen^1^, Min Zhang^1^, Jian Wu^1^, Shunliang Gao^1^, Jun Yu^1^, Risheng Que^1^, Yun Zhang^1^, Fuchun Yang^1^, Weiliang Xia^1^, Aibin Zhang^1^, Xiaofeng Tang^1^, Xueli Bai^1, 2, 3, 4 *^, Tingbo Liang^1, 2, 3, 4 *^

Correspondence to: liangtingbo@zju.edu.cn; shirleybai@zju.edu.cn

**This PDF file includes:**

Figures. S1 to S3

Tables S1 to S4

Figure. S1.

(a)Staining of tertiary lymphoid structures. CD20 (white), CD3 (red), CD4 (green), the image on the far right is the tricolor overlay image. Scale bar, 100 μm. (b) Representative IMC images and corresponding H&E images. Scale bar, 100 μm.

Figure. S2.

CN analysis of the three groups. (a) Schematic diagram of CN analysis. (b) Heatmap of the main cell clusters in each CN type. (c) Left: CN map; Right: IMC image. CD20 (cyan), CD4 (blue), CD3 (red), Pan-cytokeratin (green), CD31 (yellow), white dashed circle indicating tertiary lymphoid structures, white arrows pointing to blood vessels. Scale bar, 100 μm. (d) CN statistical graph enriched in liver cancer cells. (e) CN statistical graph enriched in macrophages and fibroblasts. (f) Statistical graph of TLS-CN. (g) CN statistical graph enriched in neutrophils. (h) CN statistical graph enriched in resident macrophages.


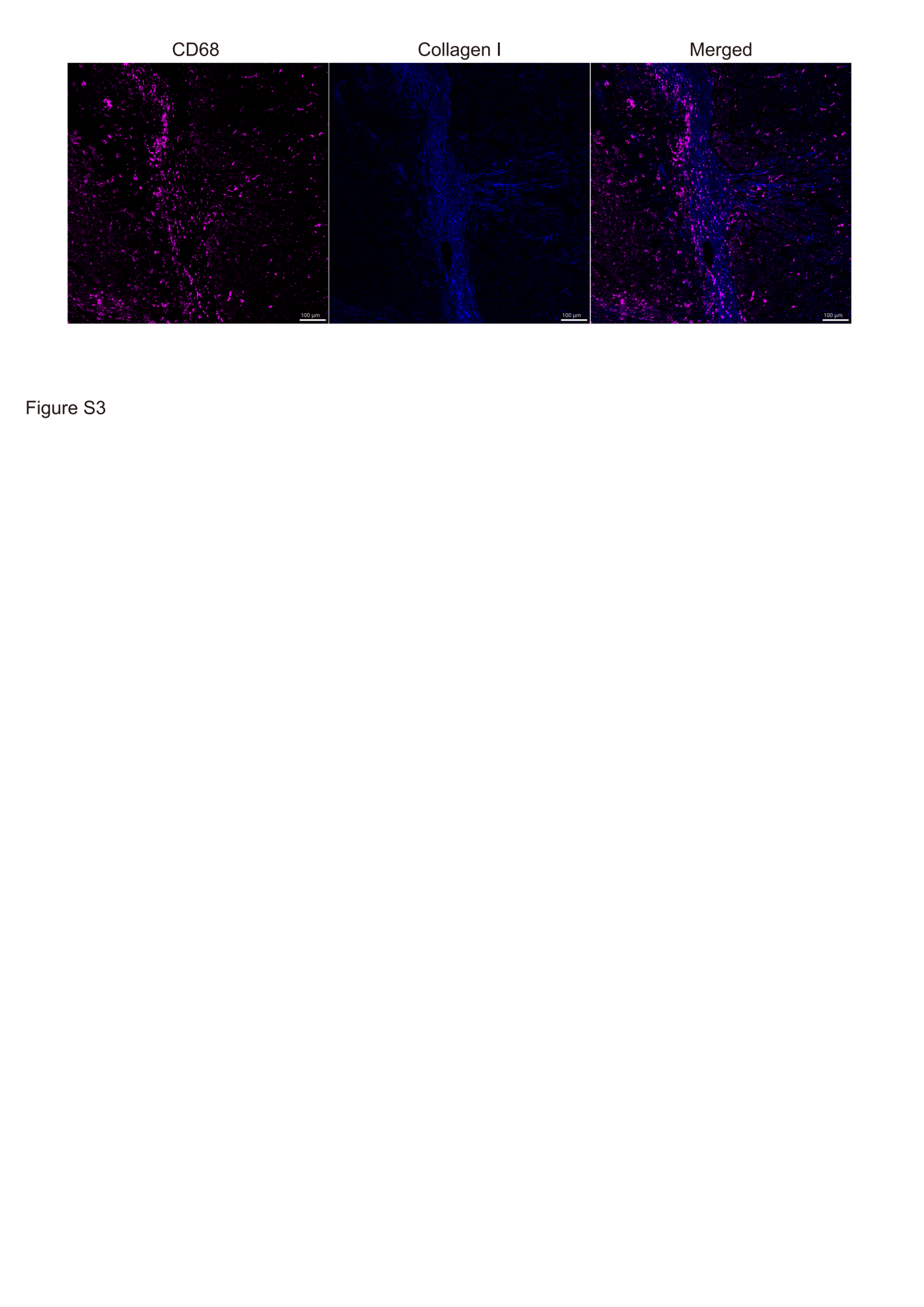


Figure. S3.

Relationships between fibroblasts and macrophages. The left image is a single staining of CD68 (magenta), marking macrophages. The middle image is a single staining of Collagen I (blue), marking fibroblasts. The right image is a composite image showing the spatial relationship between macrophages and fibroblasts. Scale bar, 100 μm.

Table S1.

Treatment efficacy

|  | **RECIST 1.1**  **(n=36)** | | **mRECIST**  **(n=36)** | |  |
| --- | --- | --- | --- | --- | --- |
| Best objective response, n (%) |  |  |  |  | |
| Complete response | 0 |  | 2 (5.6%) |  | |
| Partial response | 18 (50%) |  | 28 (77.8%) |  | |
| Stable disease | 12 (33.3%) |  | 0 |  | |
| Progressive disease | 6 (16.7%) |  | 6 (16.7%) |  | |
| Objective response rate†, n (%; 95%CI) | 18 (50%; 32.9-67.1%) |  | 30 (83.3%; 67.2-93.6%) |  | |
| Disease control rate‡, n (%; 95%CI) | 30 (83.3%; 67.2-93.6%) |  | 30 (83.3%; 67.2-93.6%) |  | |
| Duration of response, months (95%CI) | 6.8 (2.77-NA) |  | 8.12 (4.9-NA) |  | |
| Progression-free survival, months (95%CI) | 7.58 (5.1-16.1) |  | 7.58 (5.1-16.1) |  | |

^†^Objective response rate=complete response plus partial response.

^‡^Disease control rate=complete response, partial response, plus stable disease.

Abbreviations: RECIST 1.1, Response Evaluation Criteria in Solid Tumors; mRECIST, Modified Response Evaluation Criteria in Solid Tumors; CI, confidence interval; NA, not available.

Table S2.

Full eligibility criteria.

| **Inclusion criteria** |
| --- |
| Sign a written informed consent prior to the enrollment. |
| Age of 18-75 years old. |
| Hepatocellular carcinoma (HCC) confirmed by imaging, histology, or cytology (based on the American Association for the Study of Liver Diseases criteria). |
| Patients with BCLC stage C or B unresectable HCC (either with portal tumor emboli including VP1-3, but not enrolled if the main portal tumor embolus completely obstructed blood vessels and had no blood flow passage); confirmed uHCC by the experienced hepatobiliary pancreatic (HBP) multidisciplinary team, which has been established by our center since 2007; |
| At least one measurable lesion according to RECIST version 1.1 criteria (long diameter of non-lymph node lesion by CT scan ≥10 mm, short diameter of lymph node lesion by CT scan ≥15 mm). |
| No previous anti-tumor systemic therapies, including but not limited to immunotherapy, targeted therapy, anti-tumor Chinese medicine therapy, etc. |
| ECOG PS of 0-1. |
| Child-Pugh score ≤7. |
| Adequate organ functions: |
| Hematological functions: absolute neutrophil count (ANC) of 1.5×10^9^/L; platelet (PLT) ≥70×10^9^/L; hemoglobin (HGB) ≥90 g/L. |
| Liver functions: total bilirubin (TBIL) ≤1.5×upper limit of normal value (ULN); alanine aminotransferase (ALT) and aspartate transferase (AST) ≤3×ULN; serum albumin (ALB) ≥28 g/L; alkaline phosphatase (ALP) ≤5×ULN. After routine liver protection treatment, the patients who could meet the above criteria and be stable for at least 1 week were enrolled. |
| Renal functions: creatinine (Cr) ≤1.5×ULN, or creatinine clearance rate (Ccr) ≥50 mL/min. |
| Coagulation: international normalized ratio (INR) ≤1.5/prothrombin time (PT) ≤1.5×ULN，aPTT ≤1.5×ULN. If the patients were receiving anticoagulant therapy, patients with PT and INR within the prescribed range of anticoagulants were eligible. |
| Estimated survival period of ≥3 months. |
| **Exclusion criteria** |
| Fibrolamellar hepatocellular carcinoma, sarcomatoid hepatocellular carcinoma, cholangiocarcinoma, and others previously diagnosed by histology/cytology. |
| Concomitant with or history of other malignancies. |
| Previous anti-tumor systemic therapies. |
| Tumor burden exceeding 70% of the whole liver. |
| Current liver transplant candidates or individuals with a history of liver transplantation. |
| Risk of bleeding, coagulation dysfunction, or thrombolytic therapy currently; or bleeding from esophageal or gastric varices within the 6 months before enrollment. |
| Main trunk portal vein tumor thrombus (Vp4). |
| Known previous allergy to macromolecular protein preparations or applied drug components. |
| Active or a history of autoimmune disease (including but not limited to: autoimmune hepatitis, interstitial pneumonia, uveitis, enteritis, hypophysitis, vasculitis, nephritis; abnormal thyroid function (hyperthyroidism/decreased thyroid function) that could not be maintained within the normal range with medication, or a history of thyroid surgery requiring long-term thyroid hormone replacement therapy; patients with vitiligo or childhood asthma that has completely resolved without the need for any intervention in adulthood could be included, while patients with asthma requiring bronchodilators for medical intervention could not be included. |
| Patients currently using immunosuppressive agents, or systemic or absorbable local hormone therapy for immunosuppressive purposes (dose >10 mg/day prednisone or other equivalent hormones), and still continuing the use within 2 weeks before enrollment. |
| Patients using traditional Chinese medicine or other immunomodulators within 2 weeks before enrolment. |
| Symptomatic ascites or pleural effusion that could not be controlled with medication and required therapeutic puncture or drainage. |
| Poorly controlled clinical symptoms or diseases of the heart, such as heart failure above NYHA level 2, unstable angina, myocardial infarction occurred within 1 year, clinically significant supraventricular or ventricular arrhythmias requiring treatment or intervention. |
| Active or poorly clinically controlled severe infections. Severe infections within 4 weeks before the first administration, including but not limited to hospitalization due to complications of infection, bacteremia, or severe pneumonia; or fever of unexplained occurrence >38.5℃ during the screening period and before the first administration (patients could be enrolled for fever caused by the tumor, based on the investigator's judgment). |
| Past and current objective evidence of pulmonary fibrosis, interstitial pneumonia, pneumoconiosis, radiation pneumonia, drug-related pneumonia, severely impaired lung function, etc.; known syphilis infection requiring treatment; active tuberculosis (TB) currently receiving anti-tuberculosis treatment, or having received anti-tuberculosis treatment within the past year before the first administration. |
| Congenital or acquired immune deficiencies, such as HIV infection, or active hepatitis (transaminase did not meet the inclusion criteria: hepatitis B reference: HBV DNA ≥2000 IU/mL or ≥10^4^ copies/mL; hepatitis C reference: HCV RNA ≥2000 IU/mL or ≥10^4^ copies/mL; eligible for inclusion if viral load is below the above-mentioned criteria after nucleoside analogue antiviral treatment); chronic hepatitis B virus carriers with HBV DNA < 10^4^ IU/mL were eligible for inclusion, provided that they undergo antiviral treatment throughout the study. |
| Live vaccines during the study or within less than 4 weeks before the enrollment. |
| A history of psychotropic drug abuse, alcohol abuse, or drug abuse. |
| The investigator determined that other conditions should be excluded.  For example, as determined by the investigator, patients have other factors that could lead to premature termination of this study, such as other serious diseases (including mental illness) requiring combined treatment, serious laboratory abnormalities, accompanied by family or social factors that could affect the safety of patients or the collection of data and samples. |
| Central nervous system metastases. |

**Abbreviations:** HCC, hepatocellular carcinoma; BCLC, Barcelona Clinic Liver Cancer; RECIST, Response Evaluation Criteria in Solid Tumors; CT, computed tomography; ECOG PS, Eastern Cooperative Oncology Group performance score; NYHA, New York Heart Association; HBV DNA, hepatitis B virus deoxyribonucleic acid; HCV RNA, hepatitis C virus ribonucleic acid.

Table S3.

Detailed dose modification criteria

| **Dose modification criteria for envafolimab** | | |
| --- | --- | --- |
| **Immune-related AEs (irAEs)** | **Severity** | **Treatment** |
| Pneumonia | Grade 2 | Dose interruption until AEs resolved to grade 0-1 |
|  | Grade 3 or 4 or recurrent grade 2 | Permanent discontinuation |
| Diarrhea and colitis | Grade 2 or 3 | Dose interruption until AEs resolved to grade 0-1 |
|  | Grade 4 | Permanent discontinuation |
| Hepatitis | Grade 2, aspartate aminotransferase (AST) or alanine aminotransferase (ALT) >3× to ≤5× upper limit of normal (ULN), and/or total bilirubin (TBIL) >1.5× to ≤3×ULN | Dose interruption until AEs resolved to grade 0-1 and prednisone ≤10 mg/ day or equivalent dose |
|  | Grade 3, AST or ALT 5×-20×ULN and/or TBIL 3×-10×ULN | Permanent discontinuation |
|  | Grade 4, AST or ALT >20×ULN and/or TBIL >10×ULN |  |
| Myocarditis | Grade 1 | Dose interruption |
|  | ≥Grade 2 | Permanent discontinuation |
| Thrombocytopenia | Grade 3 | Dose interruption until AEs resolved to grade 0-1 |
|  | Grade 4 | Permanent discontinuation |
| Nephritis and renal dysfunction | Grade 2, creatinine >2-3×ULN | Dose interruption until AEs resolved to grade 0-1 |
|  | Grade 3, creatinine >3×ULN or >4.0 mg/Dl indicating hospitalization | Permanent discontinuation |
|  | Grade 4, life-threatening indicating dialysis treatment |  |
|  | ≥Grade 2 hyperthyroidism | Dose interruption until symptoms improved or resolved to a baseline status |
|  | ≥Grade 2 hypothyroidism | Continued medication and controlled with hormone replacement therapy |
|  | ≥Grade 2 hyperglycemia or type 1 diabetes | Dose interruption; insulin replacement therapy according to clinical needs; use of hypoglycemic drugs for hyperglycemia |
|  | Grade 2 hypophysitis | Dose interruption until the subject was clinically stable |
|  | Grade 3 or 4 hypophysitis | Dose interruption and hormone replacement therapy until the subject was clinically stable |
|  | ≥Grade 2 adrenal insufficiency | Dose interruption until the subject was clinically stable |
| Cutaneous AEs | Grade 3, or suspected Stevens-Johnson syndrome (SJS) or toxic epidermal necrolysis (TEN) | Dose interruption until AEs resolved to grade 0-1 |
|  | Grade 4, or confirmed SJS or TEN | Permanent discontinuation |
| Other irAEs | Grade 1 encephalitis | Dose interruption until AEs resolved to grade 0-1; continuation of medication for encephalitis should be based on clinical judgment |
|  | Grade 2 pancreatitis |  |
|  | Grade 2 myasthenia gravis |  |
|  | Grade 3 or 4 serum amylase increased or lipase increased |  |
|  | Other grade 2 or 3 irAEs that occurred for the first time |  |
|  | ≥Grade 2 encephalitis | Permanent discontinuation |
|  | Grade 3 or 4 pancreatitis |  |
|  | Grade 3 or 4 myasthenia gravis |  |
|  | Guillain-barre syndrome (GBS) |  |
|  | Other grade 4 irAEs that occurred for the first time |  |
| Recurrent or persistent AEs | Recurrent grade 3 or 4 (excluding endocrine disease) within 12 weeks after the last dose: grade 2 or 3 AEs did not improve to grade 0-1 or baseline status (excluding endocrine disease) or the corticosteroid dose cannot be reduced to ≤10 mg/day or equivalent | Permanent discontinuation |
| **Dose modification criteria for lenvatinib** | | |
| **AEs** | **Severity** | **Treatment** |
| Hypertension | Grade 3 (despite the best antihypertensive treatments) | Dose interruption until AEs resolved to grade 0-2 |
|  | Grade 4 | Permanent discontinuation |
| Proteinuria | ≥2 g/24h | Dose interruption until <2 g/24h |
| Nephrotic syndrome | - | Permanent discontinuation |
| Renal insufficiency or kidney failure | Grade 3 | Dose interruption until AEs resolved to grade 0-1 or baseline |
|  | Grade 4* | Permanent discontinuation |
| Cardiac dysfunction | Grade 3 | Dose interruption until AEs resolved to grade 0-1 or baseline |
|  | Grade 4 | Permanent discontinuation |
| Posterior reversible encephalopathy syndrome (PRES)/ reversible posterior encephalopathy syndrome (RPES) | Any grade | Dose interruption; restarting treatment at a reduced dose could be considered if AEs resolved to grade 0-1 |
| Hepatotoxicity | Grade 3 | Dose interruption until AEs resolved to grade 0-1 or baseline |
|  | Grade 4* | Permanent discontinuation |
| Arterial thromboembolism | Any grade | Permanent discontinuation |
| Bleeding | Grade 3 | Dose interruption until AEs resolved to grade 0-1 |
|  | Grade 4 | Permanent discontinuation |
| Gastrointestinal perforation or gastrointestinal fistula | Grade 3 | Dose interruption until AEs resolved to grade 0-1 or baseline |
|  | Grade 4 | Permanent discontinuation |
| Non-gastrointestinal fistula | Grade 4 | Permanent discontinuation |
| prolonged QT interval | >500 ms | Dose interruption until resolved to <480 ms or baseline |
| Diarrhea | Grade 3 | Dose interruption until AEs resolved to grade 0-1 or baseline |
|  | Grade 4 (despite medical management) | Permanent discontinuation |
| **Persistent and intolerable grade 2 or 3 AEs^a^ (dose reduction for lenvatinib)** | | |
| **AEs and treatment** | **Adjusted dose (body weight ≥60 kg)****^b^** | **Adjusted dose (body weight <60 kg) ^b^** |
| Dose interruption until AEs resolved to grade 0-1 or baseline^d^ when AEs occurred for the first time^c^ | 8 mg orally once daily | 4 mg orally once daily |
| Dose interruption until AEs resolved to grade 0-1 or baseline^d^ when the same or new AEs occurred for the second time | 4 mg orally once daily | 4 mg orally once every other day |
| Dose interruption until AEs resolved to grade 0-1 or baseline^d^ when the same or new AEs occurred for the third time | 4 mg orally once every other day | Permanent discontinuation |
| Permanent discontinuation of lenvatinib when life-threatening grade 4 AEs^e^ | | |

*If it is judged to be non-life-threatening with grade 4 AEs, it could be treated as a grade 3 AEs.

^a^AEs such as nausea, vomiting, and diarrhea should be actively treated before dose interruption or reduction of lenvatinib.

^b^Based on the previous dose level, the dose is gradually reduced in the order of 12, 8, 4 mg once daily or 4 mg once every other day.

^c^No dose modification for lenvatinib when hematological AEs or proteinuria occurred for the first time.

^d^Restarting treatment of lenvatinib if hematological AEs or proteinuria revolved to grade 2.

^e^If it is judged to be non-life-threatening grade 4 AEs, it could be treated as grade 3 AEs.

**Table S4.**

Comparison of similar clinical trials

| **Trial** | **Design** | **Patient** | **Arm** | | **Outcome** | **Sample size** | | **Results** | **Reference** |
| --- | --- | --- | --- | --- | --- | --- | --- | --- | --- |
| IMbrave150 | Phase III  Randomized |  | | A+B/S | OS/PFS by RECIST1.1 | 501 | | A+B (OS:19.2 m/PFS: 6.9 m)  S (OS:13.4 m/PFS:4.3 m) | PMID: 34902530 |
| LAUNCH | Phase III  Randomized | advanced primary HCC | LEN+TACE/LEN | | OS/PFS/ORR by RECIST1.1 | 338 | | LEN+TACE (OS:17.8 m/PFS:10.6 m/ORR:45.9%)  TACE (OS:11.5 m/PFS:6.4 m/ORR:20.8%) | PMID: 35921605 |
| EMERALD-1 | Phase III  Randomized |  | D+B+TACE/D+TACE/TACE | | PFS | 616 | | D+B+TACE (PFS: 15 m)  TACE (PFS: 8.2 m) | JCO.2024.42.3/LBA  432 |
| CHANCE 001 | Retrospective study | advanced HCC | PD1/L1+MTT+TACE/TACE | | PFS/ORR by mRECIST | | 456 | PD1/L1+MTT+TACE (PFS:9.5 m/ORR:60.1%)  TACE (PFS:8 m/ORR:32%) | PMID: 36750721 |
| This study | Phase II | uHCC | TACE+envafolimab+LEN | | ORR by RECIST1.1/PFS | 38 | | TACE+envafolimab+LEN (ORR:50%/PFS:7.58 m) | N/A |
