# Supplementary figures and images for: Envafolimab plus lenvatinib and transcatheter arterial chemoembolization for unresectable hepatocellular carcinoma: a prospective, single-arm, phase II study
Source: Signal Transduct Target Ther. 2024 Oct 9;9:280. doi: 10.1038/s41392-024-01991-1 (PMC11464841; doi:10.1038/s41392-024-01991-1)

**a**

CD20

CD3

CD4

Merged

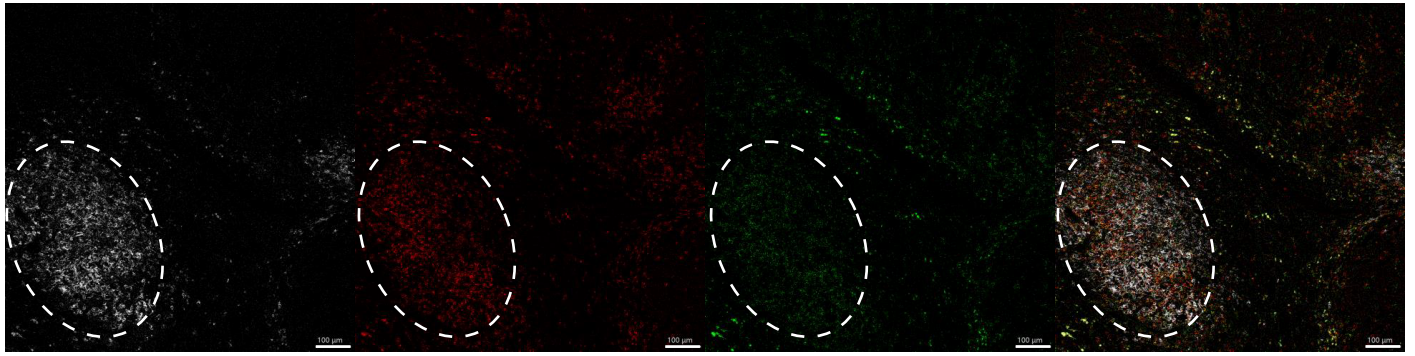**b**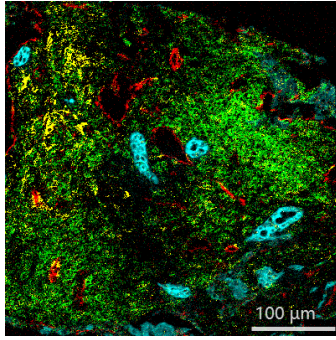

Pan-Cytokeratin CD31 αSMA CD45

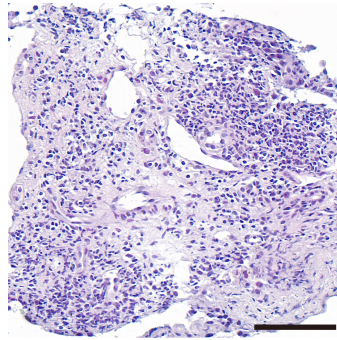

H&amp;E

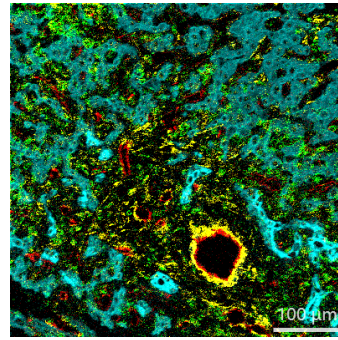

Pan-Cytokeratin CD31 αSMA CD45

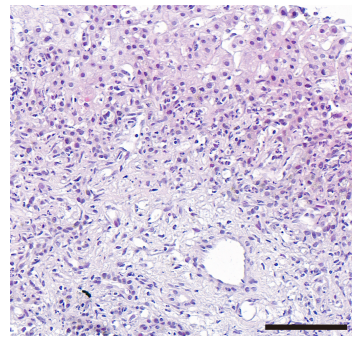

H&amp;E

Figure S1

Supplement: Supplementary file 2 — Figure S1 [file 41392_2024_1991_MOESM2_ESM.pdf]

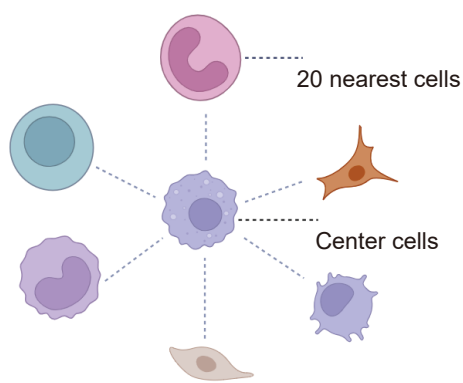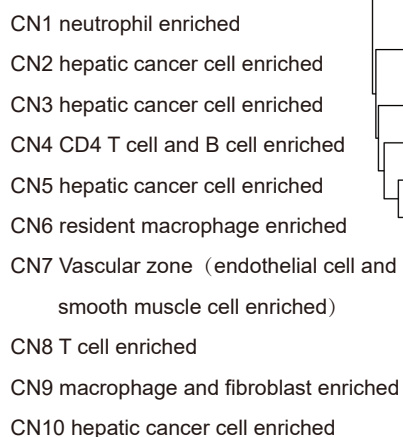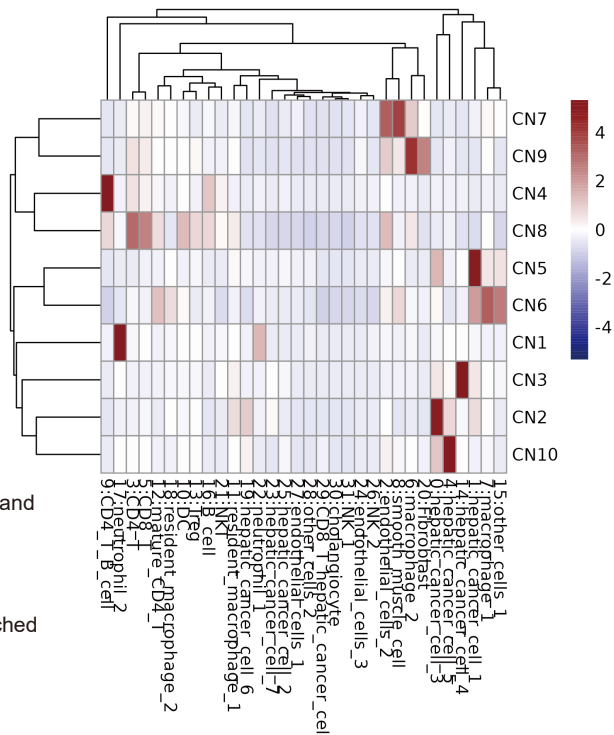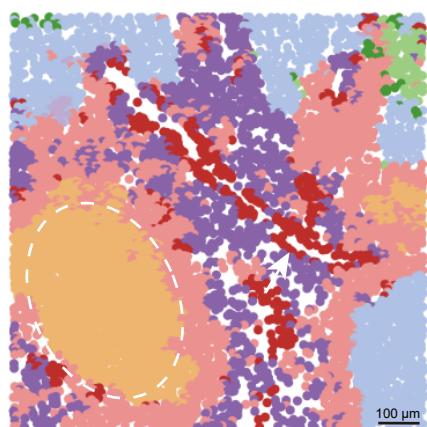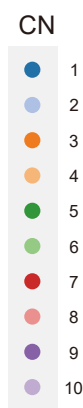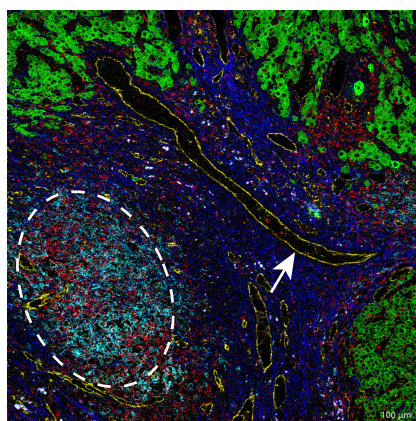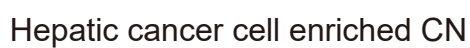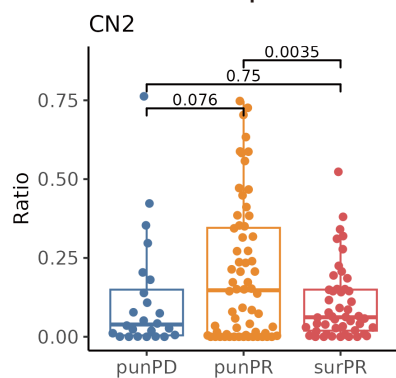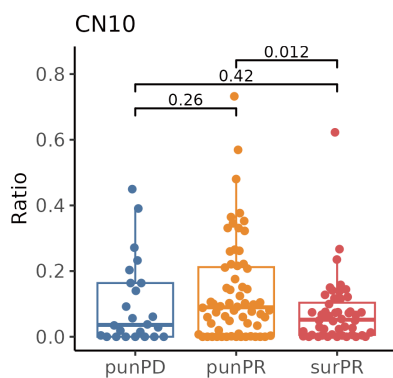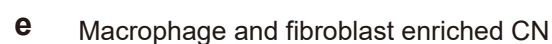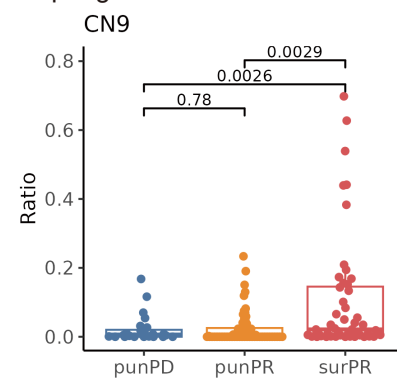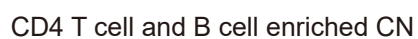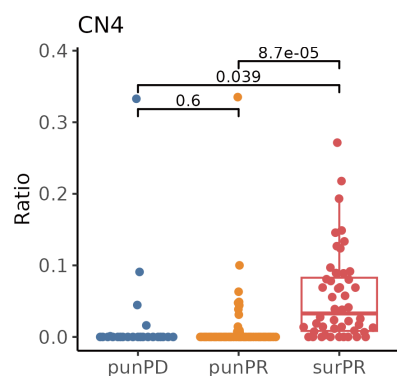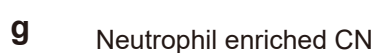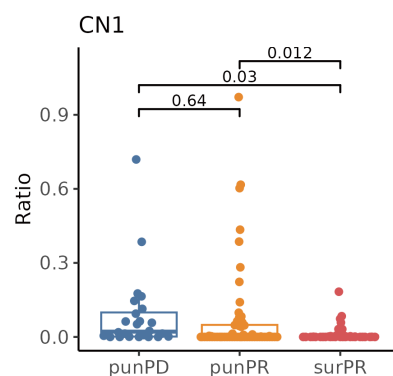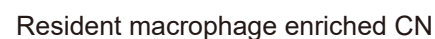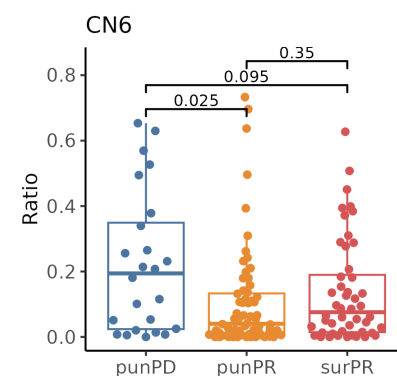

Supplement: Supplementary file 3 — Figure S2 [file 41392_2024_1991_MOESM3_ESM.pdf]

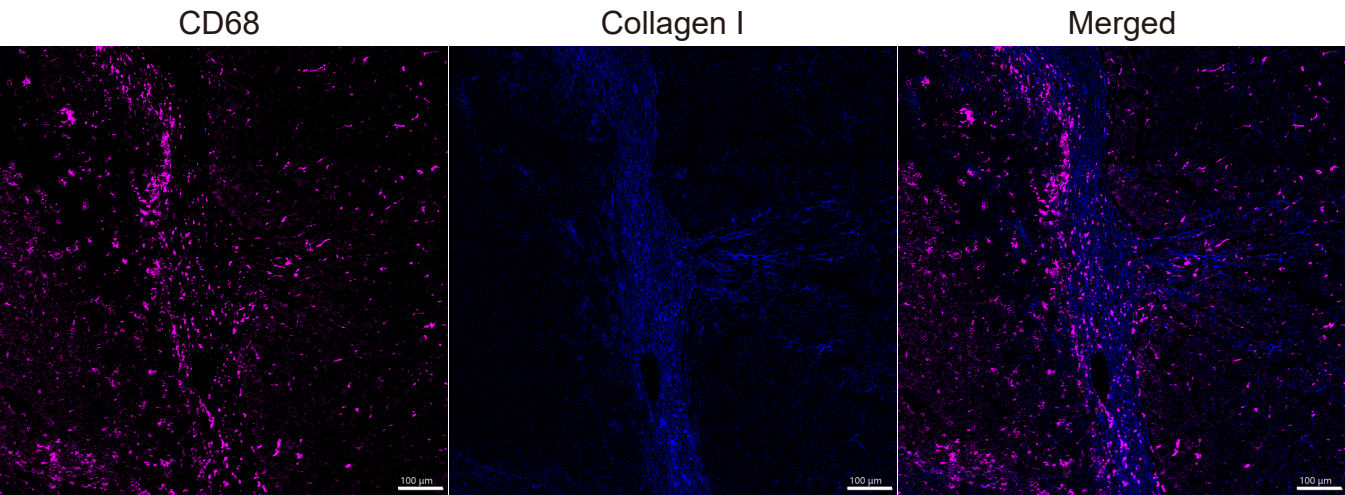

Figure S3

Supplement: Supplementary file 4 — Figure S3 [file 41392_2024_1991_MOESM4_ESM.pdf]
